# Assessment of functional improvement with temporalis myofascial flap after condylectomy in elderly patients with anterior disc displacement without reduction and an erosive condylar surface

**DOI:** 10.1186/s40902-015-0025-1

**Published:** 2015-08-12

**Authors:** Young-Hoon Kang, Jung-Suk Bok, Bong-Wook Park, Mun-Jeoung Choi, Ji-Eun Kim, June-Ho Byun

**Affiliations:** 1 Department of Oral and Maxillofacial Surgery, Institute of Health Sciences, Gyeongsang National University School of Medicine, Chilam-dong, Jinju 660-702 South Korea; 2 Department of Radiology, Institute of Health Sciences, Gyeongsang National University School of Medicine, Jinju, South Korea

**Keywords:** TMJ, Elderly patients, Disc displacement without reduction, Condylectomy, Temporalis myofascial flap

## Abstract

**Background:**

The purpose of this study was to investigate the functional effects of temporalis myofascial flap after condylectomy, with or without disc removal, in elderly patients with anterior disc displacement (ADD) without reduction and an erosive condylar surface of the temporomandibular joint (TMJ).

**Methods:**

A total of 15 joints from 11 elderly patients (71–78 years old) were included. The patients had pain, mandibular dysfunction symptoms, and unilateral or bilateral ADD as well as an erosive condylar surface of the TMJ. All patients underwent temporalis myofascial flap reconstruction after condylectomy, with or without disc removal. If the maximal mouth opening (MMO) remained <35 mm after condylectomy, coronoidotomy was also performed. Self-assessed pain and mandibular function, including MMO and protrusive and lateral movements, were evaluated.

**Results:**

No patient experienced serious complications. Most measurements improved significantly after surgery compared to preoperatively. Most patients achieved nearly-normal mouth opening at 4 weeks after surgery. Although most patients felt discomfort during active postoperative physiotherapy, no patient reported serious pain during the follow-up period.

**Conclusion:**

Although nonsurgical therapy is often the first treatment choice for ADD without reduction of the TMJ, surgical intervention involving condylectomy and temporalis myofascial flap reconstruction may be a reasonable first option for elderly patients with an erosive condylar surface of the TMJ.

## Background

Temporomandibular joint (TMJ) disorders refer to a group of conditions affecting the TMJ, masticatory muscles, and associated structures. TMJ disorders are characterized by pain, noise within the joint, limited range of motion, impaired jaw function, and closed or open jaw locking. The most common TMJ disorder is disc displacement. In most patients with this disorder, the disc is displaced anteriorly upon translation. Anterior disc displacement (ADD) without reduction of the TMJ is a widespread disorder that clinically presents itself with restricted mandibular movements, in which the morphology of the disc or condylar surface is altered. ADD leads to a greater loss of elasticity in the superior retrodiscal lamina. The disc can be forced through the discal space, eventually collapsing the joint space behind it and trapping the disc in the forward position. During mouth opening, the affected joint exhibits rotation, but translation is limited or non-existent. The articular surfaces of the bones are exposed to a greater degree of wear, which may progress to osteoarthritis in later life [1–4].

The primary goals of treatment for TMJ disorders are to increase the range of motion and relieve functional TMJ pain. Although nonsurgical therapy could be the first treatment choice for ADD without TMJ reduction, surgical interventions may be the first option in select cases, including elderly patients with an erosive condylar surface. Conservative management techniques for ADD without reduction of TMJ include physical therapy, medications, mandibular exercises and splints. There is some controversy about the treatment of ADD without reduction of the TMJ; however, a few of the conservative approaches achieve definitely satisfactory curative effects [5–7]. Magnetic resonance imaging (MRI) examinations of ADD without reduction of TMJ often show displacements of discs that are abnormally shaped and ill-remodeling or osteoarthritic changes on the condylar heads. Moreover, in many cases, the condylar surface changes, such as osteophytes and erosion, tend to be associated with long-duration symptoms and advanced ADD [3, 8].

In this study, we used a temporalis myofascial flap after condylectomy for the condylar portion of the erosive surface changes, with or without disc removal, in elderly patients with ADD without reduction and an erosive condylar surface of the TMJ. The temporalis myofascial flap is based on two dominant arterial pedicles containing the anterior and posterior deep temporal arteries. The advantages of this flap in TMJ reconstruction include its autogenous origin, adequate blood supply, and close proximity to the TMJ without the need for additional surgery. When the disc is removed, the flap can simulate physiologic action of the disc. In addition, there is minimal functional morbidity or esthetic deformity at the donor site [9, 10]. The purpose of this retrospective study was to investigate the efficacy of temporalis myofascial flap after condylectomy, with or without disc removal, in elderly patients with ADD without reduction and an erosive condylar erosive surface of the TMJ.

## Methods

In this study, we retrospectively analyzed cases involving 15 joints from 11 patients (four males, seven females) between 2010 and 2013. The inclusion criteria were patients 71–78 years old (mean age 74.3 years); maximum mouth opening <25 mm; pain and mandibular dysfunction symptoms for >4 weeks; unilateral or bilateral ADD without reduction and an erosive condylar surface of the TMJ noted on MRI; and no previous treatment attempts except analgesic medications. Patients with systemic rheumatic disease, a condylar fracture, or psychiatric disease were excluded.

### Clinical examination

The degree of pain, duration of pain and mandibular dysfunction symptoms, and presence of crepitus during mandibular movement were evaluated. Pain was determined by patient self-assessment using a visual analog scale (VAS) ranging from 0 (no pain) to 10 (worst imaginable pain). The presence or absence of crepitus was assessed during manual palpation of the lateral aspect of the TMJ. Crepitus was defined as a gravel-like, “grating” sound emanating from the TMJ during mandibular movement, which was audible to or palpable by the examiner. The clinical examination included the following parameters: determination of maximal mouth opening (MMO), as measured by the distance between the incisal edges of the maxillary and mandibular incisors; determination of the range of lateral mandibular movement, as measured by the distance between the maxillary and mandibular midline in the maximum lateral position; and determination of the range of protrusive mandibular movement, as measured by the distance from the incisal edge of the maxillary central incisor to the incisor edge of the mandibular incisor in the maximum protruded position [11].

### MRI examination

MRI examinations were performed with the 1.5-T scanner (Magnetom Avanto; Medical Solutions, Erlangen, Germany). The body coil was used as the transmitter, and two 8-cm diameter TMJ surface coils were used as the receiver. The patient was positioned supine, and the surface coil was placed directly against the patient’s TMJ region. The transection plane was scanned to identify the long axis of the condyle. Then the sagittal plane was determined to be perpendicular to this long axis, as the coronal plane paralleled the long axis The imaging protocol was as follows: (a) an axial and sagittal localizer with a repetition time (TR) of 7 ms, echo time (TE) of 2.95 ms, field of view of 26 cm, 8-mm section thickness, 179 × 256 matrix, and number of signals averaged of 2; (b) 3-mm-thick, turbo spin-echo T2-weighted (TR/TE = 3300/82), proton density (TR/TE = 3300/16), turbo spin-echo T1-weighted (TR/TE = 530/11) and gradient-echo T1-weighted (TR/TE = 450/11.2) images in the sagittal planes with the jaw closed, no intersection gap, a field of view of 14 cm, 192 × 320 matrix, and number of signals averaged of 2; (c) 3-mm-thick, proton density (TR/TE = 3000/12) and turbo spin-echo T1-weighted (TR/TE = 430/12) images in the coronal planes with the jaw closed, no intersection gap, a field of view of 18 cm, 224 × 320 matrix, and number of signals averaged of 1; and (d) 3-mm-thick, turbo spin-echo T2-weighted and proton density images in the sagittal planes with the jaw open and with the same variables as in (b). The fully open views were obtained with a 10-mm bite block in place. Maximal, comfortable mouth opening, which ranged from 3 to 4 cm, was accomplished with a notched bite block, placed between the incisors, which could be adjusted by the subject.

**Fig. 1:**
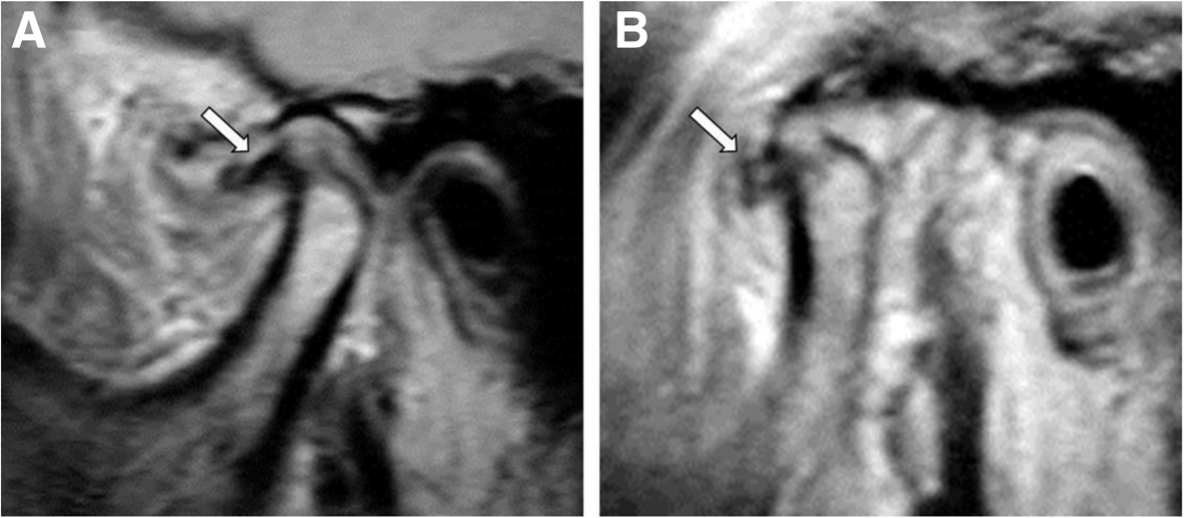
Representative MRI of ADD without reduction and an erosive condylar surface of TMJ. **a** and **b** The shape and length of the disc (*white arrows*) was also deformed in the closed and open mouth positions

In ADD without reduction, the posterior band of the disc is anterior to the superior part of the condylar head, and the intermediate zone is located anterior to the condylar head both on the closed and open mouth positions in the sagittal plane. Condylar erosion was defined as loss of continuity of the condylar articular cortex (Fig. 1) [8].

### Surgical techniques

**Fig. 2:**
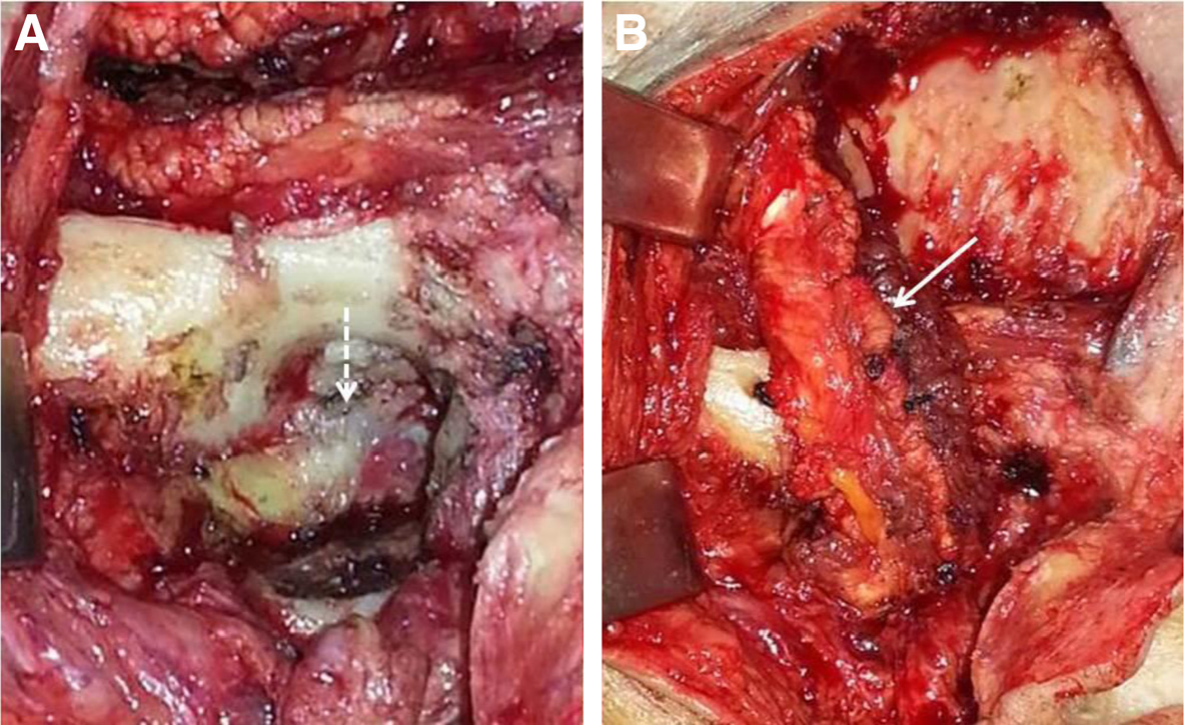
Representative anteriorly based temporalis myofascial flap. **a** Condylectomy with disc removal. **b** Outlining the temporalis myofascial flap and turning the flap into the joint space. Dotted arrow indicates the condylar space after condylectomy with disc removal and solid arrow indicates anteriorly based temporalis myofascial flap

A preauricular incision with temporal extension was made, and following dissection, a T incision was created over the joint capsule. Condylectomy with or without disc removal was performed, and a 1–1.5 cm gap was created between the condylar stump and glenoid fossa. A temporalis myofascial flap (approximately 1.5–2 cm wide and 5 cm long) was developed, which included the overlying temporalis fascia, muscle, and periosteum. The flap was then turned down over the arch and into the fossa. Drill holes into the bone of the lateral lip of the glenoid fossa were made posteriorly and anteriorly prior to placement of the flap into the joint. Turning of the flap into the joint was performed in a manner so that the fascia was facing the condyle, and the periosteum was facing the fossa (Fig. 2). If the MMO remained <35 mm after condylectomy, coronoidotomy was additionally performed via an intraoral approach. Patients were routinely administered antibiotics for a minimum of 5–7 days after surgery. Active physiotherapy began after the fifth postoperative day with jaw exercises performed at a minimal MMO of 30–35 mm. Each patient was followed for 4 weeks after surgery.

### Statistical analysis

The significance of the difference between the pre- and postoperative clinical findings was assessed with the paired *t*-test. Probabilities <0.05 were accepted as statistically significant. The statistical analysis was carried out using IBM SPSS software, version 20 (IBM Corp., NY, USA).

## Results

Fifteen joints from 11 patients with ADD without reduction and an erosive condylar surface of the TMJ were included in the study. Most of patients had a history of using analgesics. One patient used non-steroidal anti-inflammatory drug (NSAID) for approximately 12 weeks (with no breaks in analgesic consumption for >2 weeks). Palpable crepitus was present in seven patients, and one patient with bilateral TMJ involvement had crepitus on both sides. The mean preoperative VAS score was 7.7 and the mean duration of symptoms was 12.1 (range, 5–32) months (Table 1). None of the patients experienced systemic complications related to the temporalis myofasical flap. In four patients with bilateral TMJ involvement, bilateral coronoidotomy were additionally performed.

**Table 1:** Clinical findings of the patients with ADD without reduction and erosive condylar surface of TMJ

No. of patients	Side	Age (years)	Sex	VAS	Crepitus	Duration of symptom (weeks)	Period of analgestic-taking (weeks)	Condylectomy operation	Coronoidotomy operation
1	Left	74	F	8	Negative	20	12	With disc removal	-
2	Left	75	F	7	Positive	10	6	With disc removal	Ipsilateral
3	Both	73	F	9	Negative	16	8	With disc removal	Bilateral
4	Right	71	M	6	Positive	8	4	Without disc removal	-
5	Both	77	F	8	Positive	12	4	With disc removal	Bilateral
6	Both	78	F	9	Negative	12	10	With disc removal	Bilateral
7	Left	74	M	7	Positive	10	-	With disc removal	Ipsilateral
8	Right	71	F	7	Negative	8	4	Without disc removal	-
9	Left	74	M	7	Positive	5	2	With disc removal	Ipsilateral
10	Both	77	F	8	Negative	8	6	With disc removal	Bilateral
11	Left	73	F	9	Positive	12	6	Without disc removal	-

### Clinical findings

**Fig. 3:**
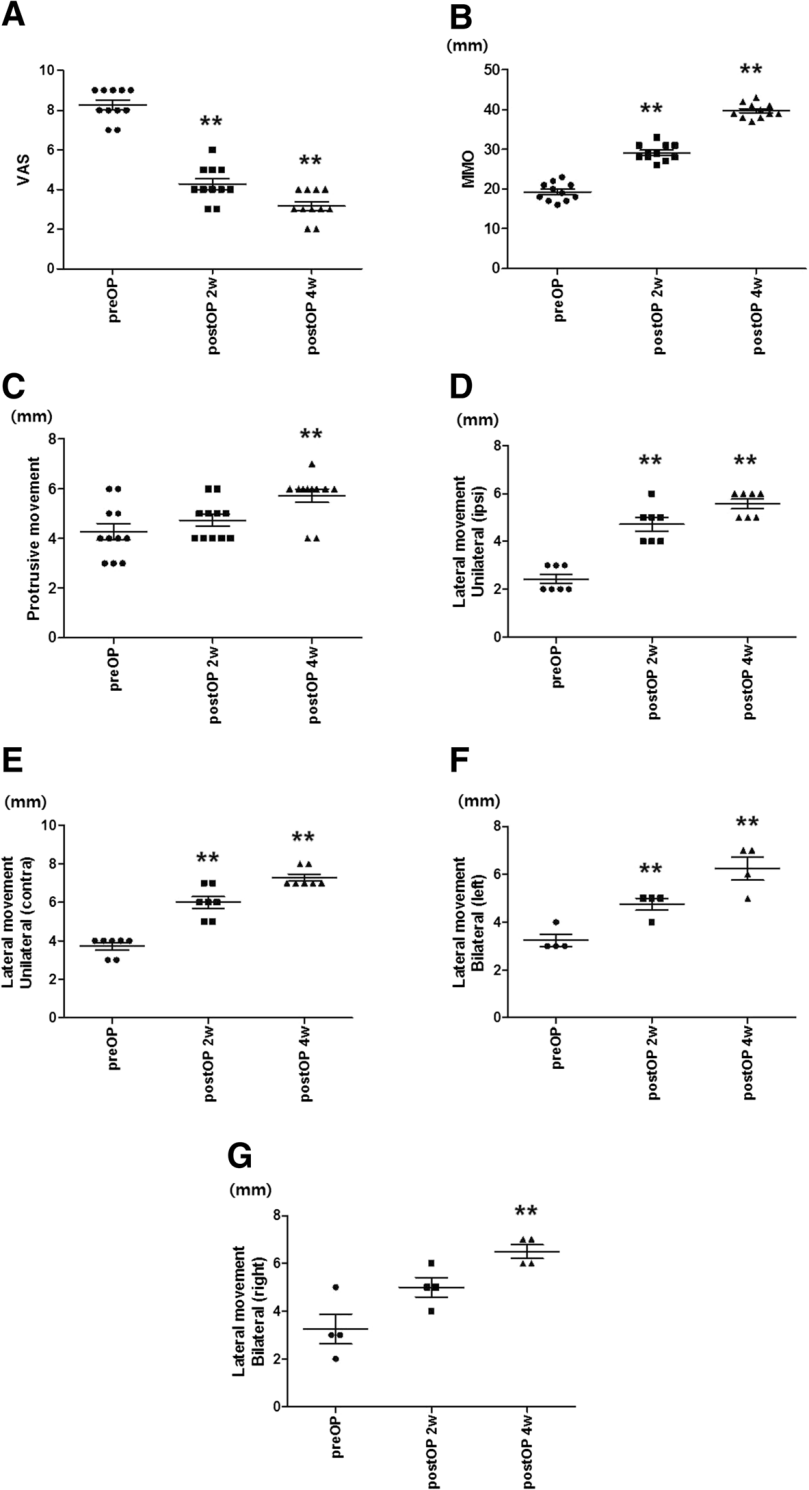
Clinical assessments of pain and mandibular function of patients postoperatively. The following were significantly improved at only 2 weeks after surgery: **a** visual analog (VAS) pain score, **b** maximal mouth opening (MMO), **d** ipsilateral lateral mandibular movement, **e** contralateral lateral mandibular movement, and **f** left lateral movement. The following were also clearly improved at 4 weeks after operation: **c** protrusive movement and **g** right lateral movement. **P < 0.01, as compared to preoperatively. preop, preoperative level; postop, postoperative level; 2W, 2 week; 4W, 4 weeks; ipsi, ipsilateral; contra, contralateral

Most measurements were significantly improved after surgery compared to preoperatively. Most patients achieved nearly-normal mouth opening 4 weeks after surgery. Although most patients felt discomfort during active physiotherapy, no patient reported serious pain during the follow-up period (Table 2). The mean preoperative VAS pain score was 7.7, whereas the mean VAS pain scores at 2 and 4 weeks postoperatively were 4.3 and 3.2, respectively. The general preoperative interincisal opening ranged from 17 to 23 mm, with a mean of 19.3 mm. At 2 weeks after surgery, the mean postoperative MMO was 29.2 mm (range, 26 to 33 mm), and at 4 weeks after surgery, the mean MMO was 39.7 mm (range, 37 to 43 mm). The mean increase in MMO from preoperative to postoperative was 9.9 mm at 2 weeks after surgery and 20.4 mm at 4 weeks after surgery. In patients with unilateral involvement, the mean preoperative ipsilateral and contralateral lateral movements were 2.4 mm and 3.7 mm, respectively. The mean postoperative ipsilateral lateral movement was 4.7 mm at 2 weeks after surgery and 5.6 mm at 4 weeks after surgery. The mean postoperative contralateral movement was 6 mm at 2 weeks after surgery and 7.3 mm at 4 weeks after surgery. In those patients with bilateral involvement, the mean preoperative left lateral movement was 3.3 mm, and the mean postoperative left lateral movement was 4.8 mm at 2 weeks after surgery and 6.3 mm at 4 weeks after surgery. The mean preoperative right lateral movement was also 3.3 mm, whereas the mean postoperative right lateral movement was 5 mm at 2 weeks after surgery and 6.5 mm at 4 weeks after surgery. The mean preoperative protrusive movement was 4.3 mm, and the mean postoperative protrusive movement was 4.7 mm at 2 weeks after surgery and 5.7 mm at 4 weeks after surgery. Compared to preoperative levels, treatment with a temporalis myofascial flap after condylectomy, with or without disc removal, significantly improved the VAS pain score, MMO, and most of the lateral mandibular movement at only 2 weeks after surgery; however, it had no effect on protrusive movement or right lateral movement in bilateral cases at 2 weeks postoperatively. All measures were clearly improved at 4 weeks after surgery (Fig. 3).

**Table 2:** Effects of temporalis myofascial flap after condylectomy with or without disc removal in the patients

Variables	Preoperative	Postoperative	
		2 weeks	4 weeks
VAS	7.7	4.3	3.2
MMO (mm)	19.3	29.2	39.7
Laterotrusion (mm)			
Unilateral			
Ipsilateral	2.4	4.7	5.6
Contralateral	3.7	6	7.3
Bilateral			
Left	3.3	4.8	6.3
Right	3.3	5	6.5
Protrusion	4.3	4.7	5.7

## Discussion

Current conservative therapies for ADD without reduction of the TMJ include patient relaxation and stress-reducing therapies; a soft diet; medications, such as analgesic agents or muscle relaxants; splints; and physiotherapy, such as ultrasound and gentle mandibular exercises. Surgical interventions include arthrocentesis, arthroscopy, and open joint surgery. Although some surgical procedures are aggressive, may lead to serious complications, and/or may be primarily reserved for patients who failed to improve following a reasonable course of nonsurgical therapy, surgery can also be used as primary treatment for patients with ADD without reduction of the TMJ.

The most common conservative therapy for ADD without reduction of the TMJ is the use of a splint. Splints can be classified into three major groups on the basis of function: stabilization splints (centric splints), distraction splints, and anterior repositioning splints. Although there are slight differences among the three kinds of splints, splint therapy generally does not reposition the disc on the condyle, although it allows the retrodiscal tissue to produce a pseudodisc where the condyle can function without limitation or pain. With splints, functional recovery of the TMJ can be obtained by adaptation of the retrodiscal tissue, without recapturing the displaced disc. Splint therapy can also decrease loading of the TMJ and remove the triggering mechanisms that program the neuromuscular system to maintain the mandible in an abnormal position [12–15]. Although several studies demonstrated that splint therapy significantly improved MMO and reduced subjective pain in patients with ADD without reduction of the TMJ, the effects of splints on ADD without reduction of the TMJ remain controversial [5, 16]. Haketa et al. [5] recently conducted a randomized clinical study evaluating the therapeutic efficacy between two treatment options for ADD without reduction: an occlusal splint and joint mobilization self-exercises. Their results suggested that joint mobilization self-exercises are an effective treatment option for improving jaw function and reducing pain and limitations of daily activities in patients with ADD without reduction. Kuboki et al. [17] reported that the elevator muscles are located behind the most posterior tooth and, therefore, the TMJ is always loaded when the elevators contract. It is usually necessary to wear splints for 6 months to 2 years, depending on the patient. Such long treatment periods can be a disadvantage in elderly patients with ADD without reduction of the TMJ. In addition, elderly patients with this condition have often had symptoms for a prolonged duration, and their disc frequently has an abnormal morphology. These factors can reduce the likelihood of achieving functional recovery of the TMJ with splint therapy. Moreover, although splints are considered one of the most effective treatment options available for ADD without reduction of the TMJ, further studies will be necessary to clarify the mechanism and efficacy of splint therapy.

Surgical intervention is another option for treatment of ADD without reduction of the TMJ. Current concepts in TMJ treatment suggest that a change in disc position is not the primary factor causing TMJ pain or dysfunction. Instead, alterations in joint pressure and a variety of biochemical constituents within the synovial fluid lead to derangement of the TMJ [18, 19]. Arthrocentesis is a simple method of flushing out the TMJ by placing needles into the superior joint space, which can be performed under local anesthesia or sedation. Arthrocentesis under sufficient pressure removes microscopic debris, pain mediators, and inflammatory cells and leads to improved movement by releasing adhesions. Although initially used to treat acute closed lock TMJ, the procedure has since developed into a treatment approach for chronic closed lock TMJ or degenerative joint disease. Several studies have reported the efficacy of arthrocentesis in treating ADD without reduction of the TMJ; however, the technique seems to be ineffective in certain conditions, such as those involving bony changes of the condyle, fibrous adhesions, and perforation of the disc [20].

TMJ arthroscopy is another type of minimally invasive surgery for treating TMJ disorders, which is usually performed under general anesthesia. Through arthroscopy, the joint can be explored, adhesions can be bluntly released or cut, and the disc can also be released. Murakami et al. [21] compared clinical short-term results of nonsurgical treatment, arthrocentesis, and arthroscopy for the management of ADD without reduction of the TMJ at 6 months after these procedures in similar age groups (mean ages of 30.4, 31.2, and 32.7 years, respectively). When the criteria for success were defined as an absence or significant reduction of pain, MMO >38 mm, and 6 mm minimum lateral and protrusive movements, the success rates were 55.6 % for the nonsurgical group, 70 % for the arthrocentesis group, and 91 % for the arthroscopy group. However, Schiffman et al. [7] reported that arthroscopy did not demonstrate statistically significant differences in effect over conservative interventions on all measured outcomes over the short- and long-term in patients (mean age, 31.8 years) with ADD without reduction. Further study is necessary to evaluate the effects of arthrocentesis and arthroscopic surgery in patients with ADD without reduction and structural alterations in the joint tissues, such as cartilage degradation and subchondral bone alterations.

Although disc repositioning with high condylectomy can be employed as an alternative method, with a high therapeutic success rate in ADD without reduction of the TMJ, disc repositioning may be an unreliable and ineffective strategy in patients with ADD without reduction of the TMJ and condylar erosion. Glycosaminoglycans, one of the main components of the TMJ, decrease significantly in patients whose symptoms have been present for a prolonged period, and these reduced levels can lead to disc degeneration. ADD without reduction of the TMJ and condylar erosion are frequently observed in patients with symptoms of a relatively long duration. Furthermore, the disc’s ability to bear heavy loads is impaired by the degeneration caused by disc displacement. Furthermore, Li et al. [22] reported that patients with ADD without reduction, particularly those in whom the disorder is bilateral, have a higher risk of rupture after repositioning the disc by arthroscopy. Therefore, disc repositioning may be considered only when the disc is minimally deformed and has a near-normal length [23–26].

## Conclusion

In the present study, significant improvement of mandibular function was obtained within the first 4 weeks after surgical treatment. Although long-term follow-up evaluation is necessary, our study suggests that a temporalis myofascial flap after condylectomy may be a valuable first-line treatment option in elderly patients with ADD without reduction and an erosive condylar surface of the TMJ.
